# Epimedin C Alleviates Glucocorticoid-Induced Suppression of Osteogenic Differentiation by Modulating PI3K/AKT/RUNX2 Signaling Pathway

**DOI:** 10.3389/fphar.2022.894832

**Published:** 2022-07-04

**Authors:** Yongxiang Xu, Shichun Chen, Linxuan Huang, Weichao Han, Yingying Shao, Minyi Chen, Yusheng Zhang, Ruirong He, Baocheng Xie

**Affiliations:** ^1^ Department of Pharmacy, Affiliated Dongguan Hospital, Southern Medical University, Dongguan, China; ^2^ Dongguan Institute of Clinical Cancer Research, Affiliated Dongguan Hospital, Southern Medical University, Dongguan, China; ^3^ Department of Pharmacy, The First People’s Hospital of Foshan (The Affiliated Foshan Hospital of Sun Yat-Sen University), Foshan, China

**Keywords:** epimedin C, PI3K, and AKT signaling pathways, osteoporosis, dexamethasone, osteogenic differentiation

## Abstract

Secondary osteoporosis is triggered mostly by glucocorticoid (GC) therapy. Dexamethasone (DEX) was reported to inhibit osteogenic differentiation in zebrafish larvae and MC3T3-E1 cells in prior research. In this research, we primarily examined the protective impacts of epimedin C on the osteogenic inhibition impact of MC3T3-E1 cells and zebrafish larvae mediated by DEX. The findings illustrated no apparent toxicity for MC3T3-E1 cells after administering epimedin C at increasing dosages from 1 to 60 μM and no remarkable proliferation in MC3T3-E1 cells treated using DEX. In MC3T3-E1 cells that had been treated using DEX, we discovered that epimedin C enhanced alkaline phosphatase activities and mineralization. Epimedin C could substantially enhance the protein expression of osterix (OSX), Runt-related transcription factor 2 (RUNX2), and alkaline phosphatase (ALPL) in MC3T3-E1 cells subjected to DEX treatment. Additionally, epimedin C stimulated PI3K and AKT signaling pathways in MC3T3-E1 cells that had been treated using DEX. Furthermore, in a zebrafish larvae model, epimedin C was shown to enhance bone mineralization in DEX-mediated bone impairment. We also found that epimedin C enhanced ALPL activity and mineralization in MC3T3-E1 cells treated using DEX, which may be reversed by PI3K inhibitor (LY294002). LY294002 can also reverse the protective impact of epimedin C on DEX-mediated bone impairment in zebrafish larval. These findings suggested that epimedin C alleviated the suppressive impact of DEX on the osteogenesis of zebrafish larval and MC3T3-E1 cells via triggering the PI3K and AKT signaling pathways. Epimedin C has significant potential in the development of innovative drugs for the treatment of glucocorticoid-mediated osteoporosis.

## Introduction

Osteoporosis is a prevalent bone illness hallmarked by reduced bone density, deterioration of bone microstructure, and decreased bone strength, leading to an elevated risk of fracture (Armas and Recker, 2012; Coughlan and Dockery, 2014). GC is extensively utilized for anti-inflammatory treatment in all kinds of immune-mediated diseases, including rheumatic arthritis, systemic lupus erythematosus, asthma, inflammatory bowel disease (Barnes, 2014; Compston, 2018). Long-term glucocorticoid medication, on the other hand, has a number of side effects. Overuse of glucocorticoid (GC) is a major cause of secondary osteoporosis, and studies have shown that up to 40% of individuals who use GCs on a regular basis are at a greater risk of fracture (Whittier and Saag, 2016). Glucocorticoids can inhibit cellular proliferation, osteogenic differentiation, and osteocyte apoptosis *in vitro and in vivo* (Wang et al., 2017; Xie et al., 2019). The GIOP-related guidelines issued by the American College of Rheumatology recommend bisphosphonates as first-line treatment for GIOP. Bisphosphonates have lower cost and significantly increase bone density in patients with osteoporosis. However, some studies have found that long-term use of bisphosphonates for osteoporosis increases the risk of atypical femoral fractures (Adler et al., 2016). As a consequence, innovative drugs to treat GC-mediated osteoporosis are urgently needed.

The therapeutic properties of epimedium are identified as having diverse pharmacological applications, including, anti-tumor, anti-diabetic, hepatoprotective, and anti-osteoporosis functions (Liu et al., 2006; Kim et al., 2017; Guo et al., 2018). Epimedin C, an ingredient of epimedium, has the potential for the treatment of cardiovascular disease and bone loss (Lee et al., 2014; Shui et al., 2017). Recent research discovered that epimedin C considerably enhanced blood perfusion and bone weight in an osteogenesis-induced murine model, as well as blood perfusion and tumor-related angiogenesis in treated 4T1 in mice with tumors (Shui et al., 2017). In another research report, it was shown that epimendin C triggered the differentiation of C3H/10T1/2 cells into endothelioid cells (Zhao et al., 2015). Epimendin C’s role in GC-mediated osteogenic inhibition, however, is uncertain.

The phosphatidylinositol 3-kinase (PI3K)/Akt signaling pathway modulates a variety of biological functions in the body, such as cell proliferation, metabolism, angiogenesis, metastasis, growth, and differentiation (Ersahin et al., 2015). Studies have also confirmed the significant modulatory function of the PI3K/Akt signaling pathway in osteoporosis, which can regulate the differentiation of osteoclasts and osteogenic differentiation (Yeon et al., 2019; Cui et al., 2020; Li and Wang, 2022). However, the modulatory function of the PI3K/Akt signaling pathway in glucocorticoid-induced osteoporosis remains to be further explored. Eucommia ulmoides, Drynaria, and Cuscuta extracts alleviate glucocorticoid-mediated osteoporosis by suppressing osteoclastogenesis via PI3K/Akt pathway (Han et al., 2021). At the same time, the study found that dexamethasone inhibited the osteogenic differentiation of osteoblasts *in vivo* and *in vitro* through the PI3K/Akt signaling pathway (Pan et al., 2019). This illustrated that stimulating the PI3K/Akt signaling pathway can relieve the glucocorticoid-mediated suppression of osteogenic differentiation. A study found that epimedin C may stimulate the PI3K/Akt pathway to alleviate Alzheimer’s disease (Sheng et al., 2020). Hence, we examined the involvement of epimedin C in the suppression of osteogenesis caused by dexamethasone (DEX) in zebrafish and MC3T3-E1 cells.

## Materials and Methods

### Cell Culture

MC3T3-E1 cells were grown in α-MEM (A1049001, Gibco) combined with 10 percent (v/v) fetal bovine serum (FBS) (10099-141, Gibco) solution, followed by incubation at 37°C in humid air that contained 5 percent CO_2_.

### Preparation of the Solution

After dissolving Epimedin C in 100 mM dimethyl sulfoxide (DMSO) at a temperature of -20°C, we subsequently diluted it using α-MEM to the designed experimental concentration.

### Assays for Cell Viability and Proliferation

The viability of the cells was evaluated utilizing Cells Counting Kit-8 (CCK-8) tests (Dojindo Laboratories, Japan). We then cultured MC3T3-E1 cells in 96-well plates (3×10^3^ cells/well). Thereafter, we concurrently cultured the cells with 10, 20, 40, 60, 80, and 100 μM epimedin C for 3 days, sequentially, to assess the toxicity of epimedin C in MC3T3-E1 cells. To evaluate the proliferation of epimedin C in DEX-mediated viability, the cells belonging to the experimental group were concurrently cultured in 10, 20, and 40 μM epimedin C correspondingly for 3 days. After the experiment, CCK-8 (10 μL) and complete medium (90 μL) were added, and cultured for 2 h in a CO_2_ incubator. The microplate reader (Thermo Fisher Scientific Inc., United States) set the wavelength of 450 nm to detect the absorbance. The digested cells were seeded in 24-well plates, and BeyoClick™ EdU-594 (C0078S, Beyotime) was used to evaluate cell proliferation after 24 h. First, diluted EdU (10 µM) was added to the cell culture medium and incubated for 2 h. Cells were fixed with 4% paraformaldehyde for half an hour and treated with 0.5% Triton X-100 for 10 min. After washing with 3% BSA, cells were stained with Click Additive Solution for 30 min at room temperature. After washing with 3% BSA, cells were incubated with Hoechst 33342 stain for 10 min. Finally, the percentage of EdU^+^ cells at five light fields/well was observed and counted using a fluorescence microscope (Olympus Corporation).

### Assays for ALP Activity and Staining

To assess the pharmacological effects of epimedin C in osteogenic differentiation, cells belonging to the experimental group were subjected to treatment using 10, and 20 μM epimedin C, correspondingly, and 10 μM DEX. The MC3T3-E1 cells’ ALP function was assessed utilizing an ALP assay kit after continuous administration and culture for 5 days. According to the instructions, the activity detection set the detection wavelength of the microplate reader to 405 nm. After fixing the cells with cell fixative for 20 min, we employed the BCIP/NBT kit (C3206, Beyotime) to conduct ALP staining.

### Mineralization Assay

Alizarin red staining was utilized to assess the mineralization nodes of MC3T3-E1 cells. The next step involved the induction of MC3T3-E1 cells (2.5×10^5^ cells/well) using an osteogenic induction medium (OM, comprising 50 μg/ml ascorbic acid, 10 mM β-glycerophosphate, and 0.1 μM DEX). Cells belonging to the experimental group were subsequently subjected to 10, and 20 μM epimedin C treatment, correspondingly, and 10 μM DEX for 21 days. This was followed by fixing of the MC3T3-E1 cells with cell fixative for 20 min, rinsing twice in PBS, and staining for 30 min utilizing alizarin red S. Alizarin red-stained mineralization nodes were photographed using an inverted microscope. The mineralization nodes stained with alizarin red S were isolated using 10 percent cetylpyridinium chloride (CPC, Sigma), and the absorbance was quantitatively detected at 562 nm wavelength.

### Western Blotting

Lysis of the MC3T3-E1 cells was achieved with the help of RIPA strong lysis buffer that contained protease inhibitors and phosphorylase inhibitors for 30 min to prepare total protein extract. We prepared a standard protein curve in accordance with the guidelines of the BCA detection kit to assess the proportions of each protein in the sample. After normalization of each sample, 30 μL of protein was introduced into 10 percent SDS-PAGE before electro-transferring them to PVDF for immunoblotting. The following antibodies were utilized for incubation: anti-OSX (1:1000, ab209484, Abcam), Anti-ALPL (1:2000, ab65834, Abcam), Phospho-Akt (Ser473) (1:2000, #4511, Cell Signal Technology), anti-RUNX2 (1:1000, ab236639, Abcam), Akt (pan) (C67E7) (1:2000, #4691, Cell Signal Technology), Phospho-PI3K p85 alpha (Tyr607) (1:1000, #AF3241, Affinity), PI3 kinase P110 alpha Antibody (1:1000, #AF5112, Affinity) and anti-β-actin antibody (1:1000, AF0003, Beyotime). Incubation of the PVDF was done at 4°C throughout the night with diluted antibodies. We subjected the PVDF to 1 h of incubation with goat anti-mouse IgG H&L (HRP) (1:5000, ab6789, Abcam) or with goat anti-rabbit IgG H&L (HRP) (1:5000, ab6721, Abcam) at 25°C. Chemiluminescence analysis using a gel imaging system (Bio-Rad, United States) after incubating proteins with ECL luminescent solution.

### Alizarin Red Staining and Mineralization Quantitative Analysis

Shanghai FishBio Co., Ltd. provided wild-type zebrafish larvae (AB strain) and zebrafish TG (ola.sp7:nlsGFP). Culturing of AB strain larvae was done within isothermal settings at 28.5°C in a medium that contained 10 ppm methylene blue, 5 mmol/L NaCl, 0.17 mmol/L KCl, 0.16 mmol/L MgSO_4_, and 0.33 mmol/L CaCl_2_. Epimedin C and dexamethasone to appropriate concentrations and dissolved them into the culture water of zebrafish larvae for administration. This was followed by staining of the zebrafish larvae on nine dpf using alizarin red S and calcein staining. Firstly, zebrafish larvae were immobilized for 2 h in 4 percent polyformaldehyde and then dried for 30 min in 50 percent ethanol. The zebrafish larvae were once again bleached for 30 min using 1 percent H_2_O_2_ and 1 percent KOH. The zebrafish larvae were then subjected to staining for 6 h with 0.01 percent alizarin red staining in 0.5 percent KOH. Subsequently, the zebrafish larvae were decolorized for a duration of 6–8 h in differing concentrations of 0.5 percent KOH and glycerin (3:1, 1:1, 1:3). The lateral and ventral views of zebrafish were photographed with the aid of a stereomicroscope. For calcein staining, the zebrafish on the nine dpf were anesthetized with anesthesia, then 0.2% calcein solution was added for 15 min, rinsed thrice using water, and photographed with an upright fluorescence microscope. We anesthetized the zebrafish TG (ola.sp7:nlsGFP) treated with anesthesia, and placed them under an upright fluorescence microscope for fluorescence photography. Image-Pro Plus (IPP, Media Cybernetics, United States) was utilized to examine the mineralized region and integrated optical density (IOD) of skull alizarin red staining.

### Statistical Analysis

The data were reported as mean ± standard deviations (SD). SPSS 17.0 was utilized to analyze statistical data. A one-way analysis of variance (ANOVA) was utilized for comparing several groups and statistical results with *p* < 0.05 were interpreted as having statistical significance.

## Results

### Impact of Epimedin C on the Viability of MC3T3-E1 Cells Viability and MC3T3-E1 Cells Treated With DEX

**FIGURE 1:**
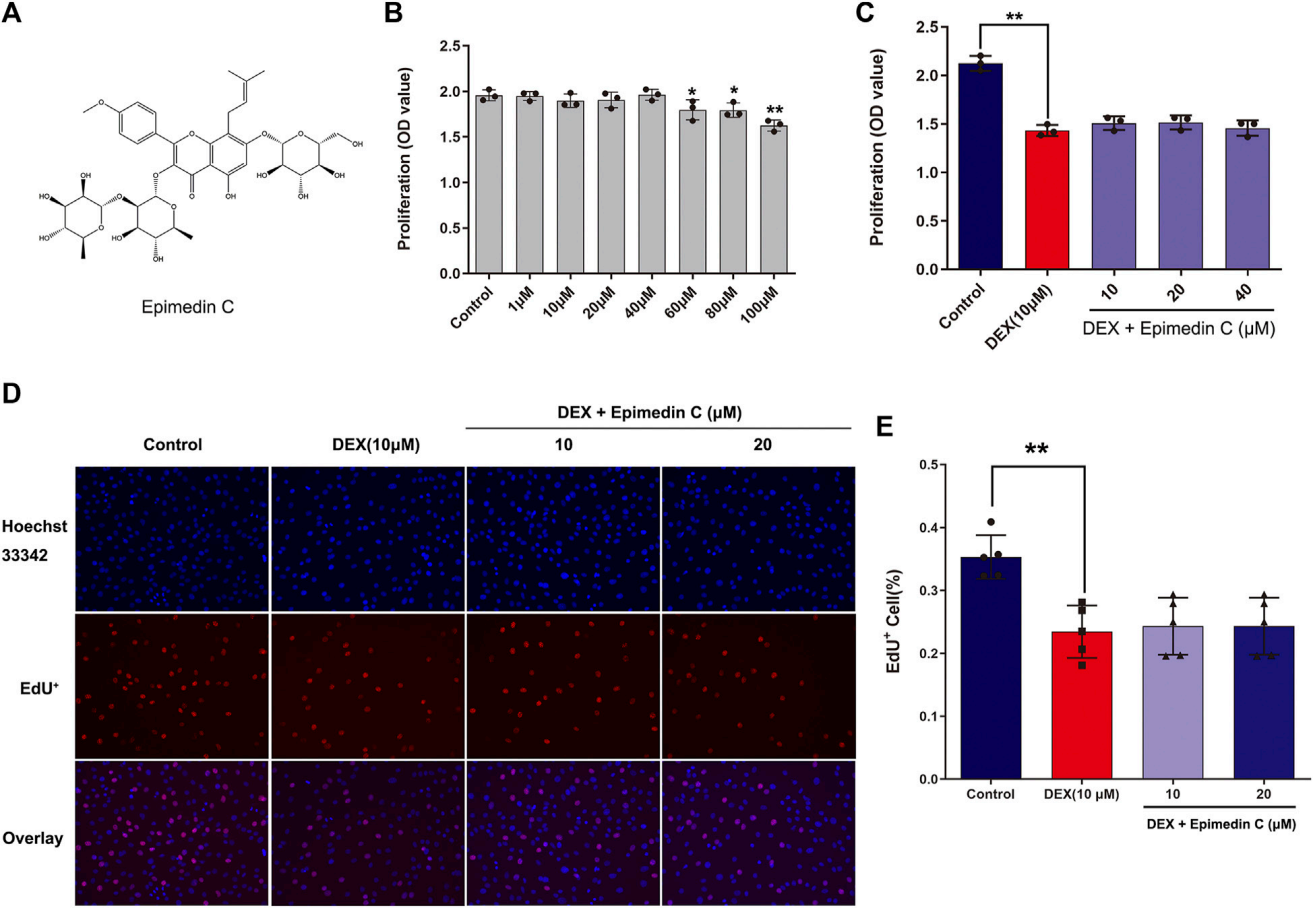
Impacts of epimedin C on MC3T3-E1 cells viability and DEX-treated MC3T3-E1 cells. **(A)** The structural formula of epimedin **(C)**. **(B)** Epimedin C was used to treat the cells for 72 h at various doses; **(C)** The MC3T3-E1 cells viability was determined utilizing CCK-8 assay after 72 h of incubation with epimedin C and DEX (10 μM). **(D,E)**: Cells were treated for 24 h with epimedin C (10 and 20 μM) and DEX. (10 μM) and analyzed by EdU assay. *p < 0.05, **p < 0.01 vs. control.

To examine the impact of epimedin C on MC3T3-E1 cells viability, the CCK-8 assay was employed. Upon administering epimedin C to MC3T3-E1 cells for 72 h at increasing dosages of 1–40 μM, the results revealed no evident toxicity (Figure 1B). However, the findings also illustrated no remarkable proliferation in MC3T3-E1 cells subjected to DEX treatment after applying epimedin C with 10 μM, 20 μM, and 40 μM (Figure 1C). The results of EdU assay showed that dexamethasone at a concentration of 10 μM could significantly inhibit cell proliferation (*p* < 0.01), while epimedin C (10 and 20 μM) had no effect on the inhibition of cell proliferation (Figures 1D, E).

### Epimedin C Ameliorated the Inhibiting Impact of MC3T3-E1 Cells Subjected to DEX Treatment on ALP Activity

**FIGURE 2:**
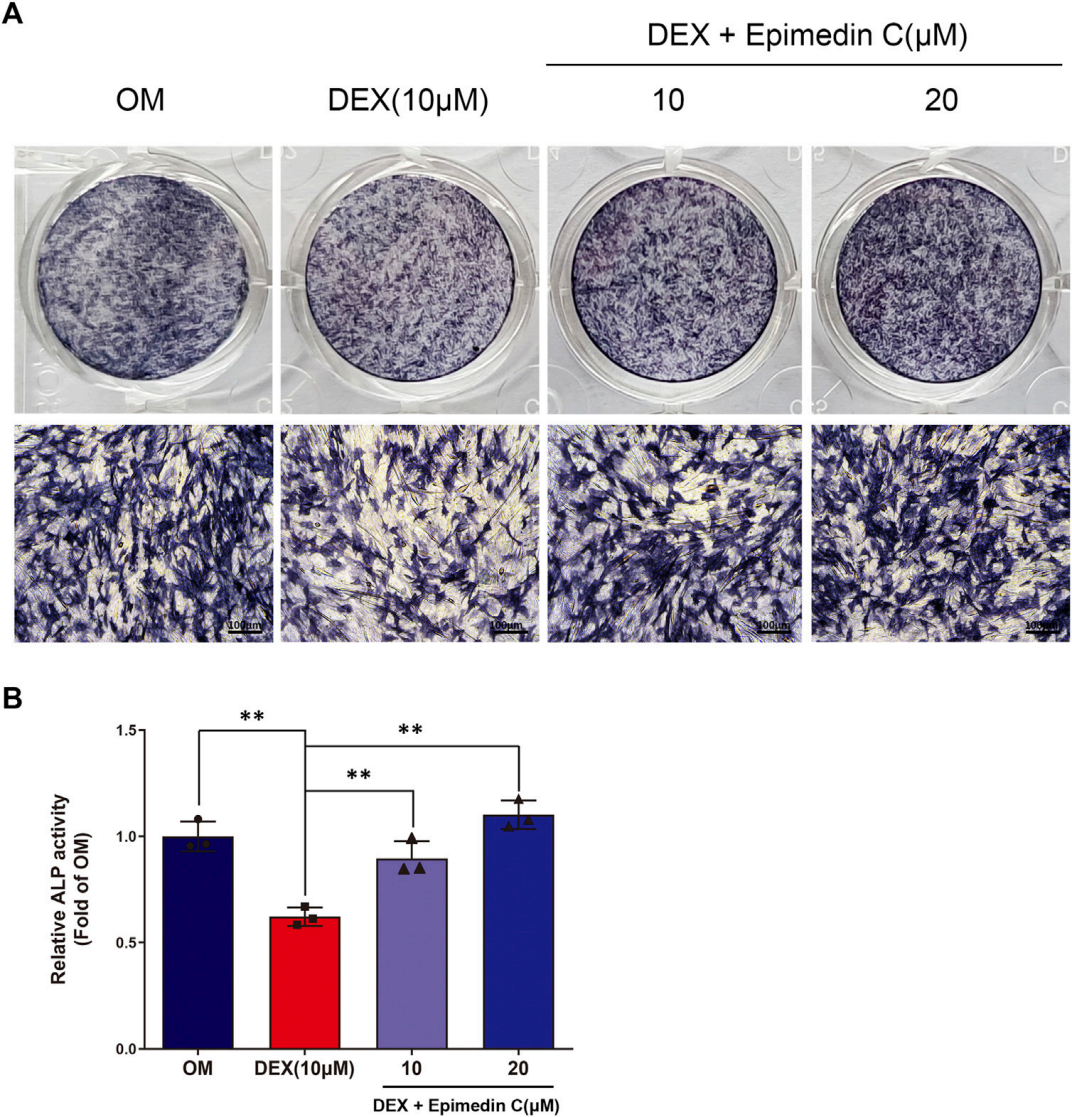
Epimedin C’s impact on the action of ALP in MC3T3-E1 cells subjected to DEX treatment. **(A)** Following MC3T3-E1 cells treatment for 5 days using epimedin C and DEX, cells were ALP stained with BCIP/NBT kit. **(B)** MC3T3-E1 cells were concurrently for 5 days treated using epimedin C (10, 20 μM) and DEX (10 μM) in OM, and the activity of ALP was determined. ^**^ *p < 0.01*.

Since ALP action in DEX-treated MC3T3-E1 cells is a near-marker of osteogenic differentiation, we investigated the impact of epimedin C on the ALP function. After being cultured in OM, the MC3T3-E1 cells were subjected to incubation for 5 days in DEX (10 μM) and epimedin C (10 μM, 20 μM). The findings of ALP staining and ALP activity illustrated that adding DEX considerably suppressed the function of ALP in MC3T3-E1 cells. Contrastingly, the inhibited ALP activity was significantly alleviated when epimedin C was added (*p* < 0.01) (Figure 2).

### Epimedin C Attenuated the Suppressive Impact of MC3T3-E1 Cells Treated With DEX on Mineralization

**FIGURE 3:**
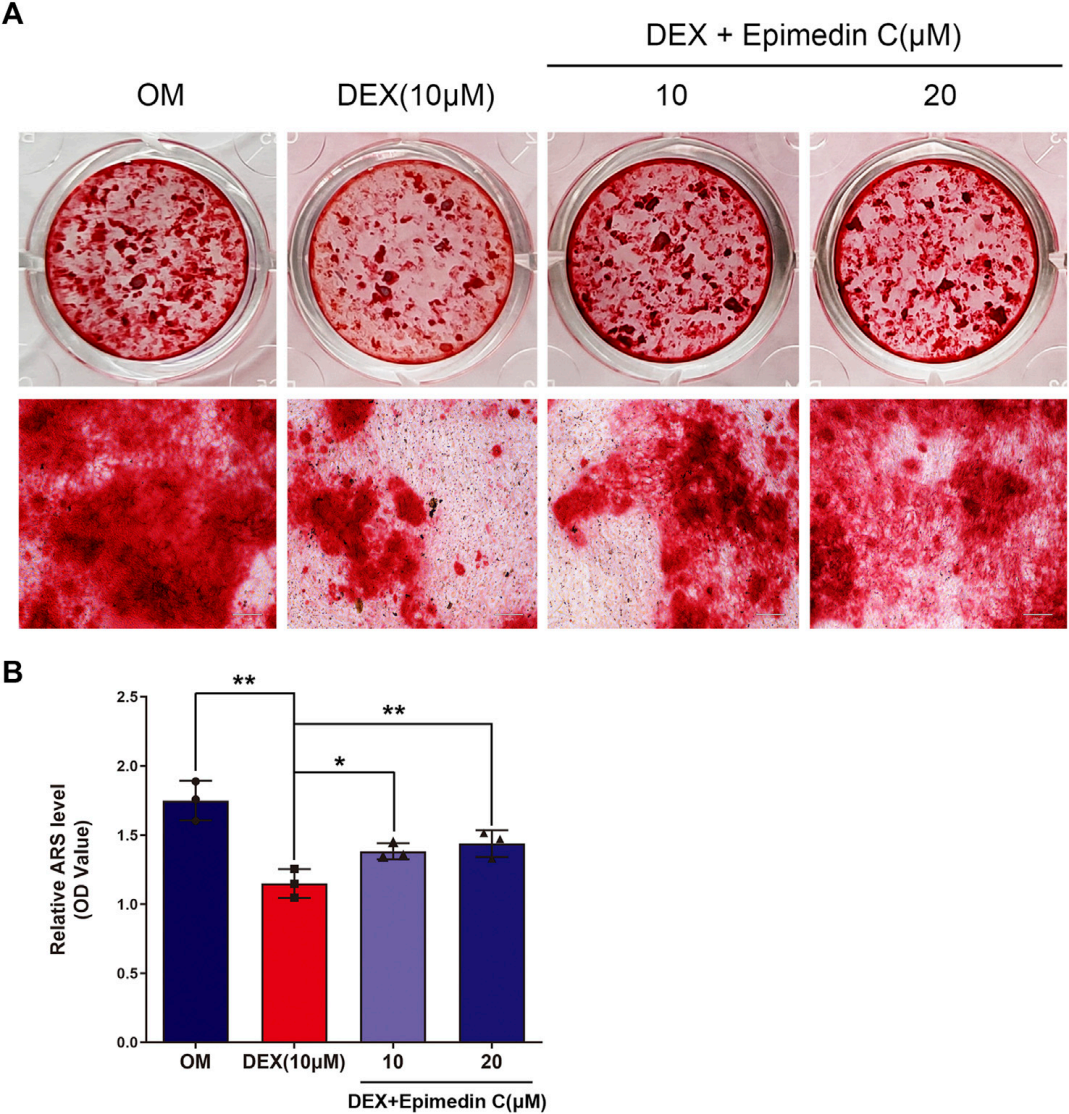
Epimedin C attenuated the inhibiting impact of DEX on mineralization in MC3T3-E1 cells. **(A)** Upon MC3T3-E1 cells treatment using epimedin C and DEX in OM for 21 days, alizarin red S was utilized for staining the mineralized nodules. **(B)** Isolation of alizarin red S dye with 10 percent CPC was carried out to quantify the mineralization status. DEX: dexamethasone, OM: osteogenic induction medium. *p < 0.05, **p < 0.01.

The MC3T3-E1 cells were cultured in OM and the cells belonging to the experimental group were subjected to incubation in DEX (10 μM) and epimedin C (10 μM, 20 μM) for 21 days. The mineralization nodes were visualized using Alizarin red staining. We found that DEX (10 μM) inhibited mineralization nodes of MC3T3-E1 cells in contrast with OM. Nevertheless, the inhibited effect was significantly alleviated when epimedin C was added (*p* < 0.01) (Figure 3).

### Epimedin C Promoted the OSX, RUNX2, and ALPL Protein Expression in MC3T3-E1 Cells Subjected to DEX Treatment

**FIGURE 4:**
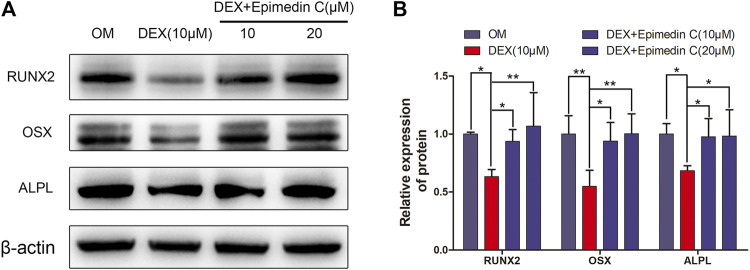
Epimedin C enhanced the OSX, RUNX2, and ALPL protein expression in MC3T3-E1 Cells treated with DEX. **(A)** Following 7 days of MC3T3-E1 cells treatment using epimedin C and DEX in OM, the protein was extracted by 10 percent SDS-PAGE and identified using the specified antibodies of OSX, RUNX2, and ALPL. As a loading control, β-actin was employed. **(B)** The bar charts depicted ImageJ’s measurements of RUNX2, OSX, and ALPL. DEX: dexamethasone; OM: osteogenic induction medium. ^*^ *p* < 0.05, ^**^ *p* < 0.01.

In order to explore the impacts of epimedin C on osteogenesis-related proteins, we extracted protein for 7 days. The findings demonstrated that the expression levels of OSX, RUNX2, and ALPL protein were substantially reduced in the DEX group, whereas the epimedin C group with 10 and 20 μM reduced the inhibitory impacts of DEX and considerably elevated the RUNX2, OSX, and ALPL protein expression levels in contrast with DEX group (Figure 4).

### Epimedin C Stimulated the PI3K-AKT Signaling Pathway in MC3T3-E1 Cells Subjected to DEX Treatment

**FIGURE 5:**
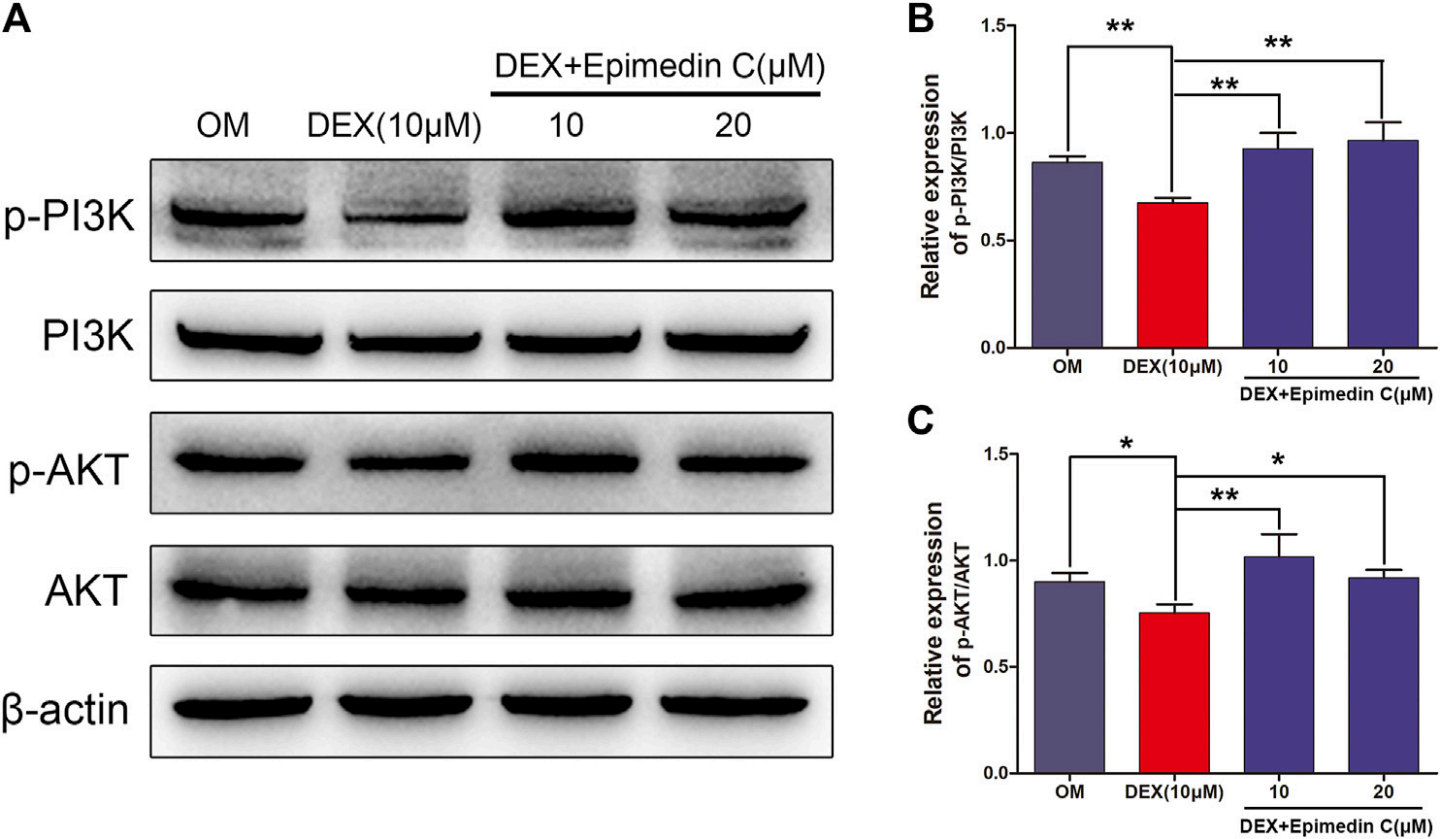
Epimedin C activated AKT and PI3K phosphorylation in MC3T3-E1 cells treated using DEX. **(A)** Western blotting was employed to assess the proportions of phosphorylated PI3K and AKT. As an endogenous control, β-actin was employed. **(B,C)**: ImageJ was used to quantify the p-PI3K/PI3K and p-AKT/AKT ratios as depicted by the bar charts. DEX: dexamethasone, OM: osteogenic induction medium. ^*^ *p* < 0.05, ^**^ *p* < 0.01.

The PI3K-AKT signaling pathway is critical for osteogenic differentiation. In DEX-treated MC3T3-E1 cells, we performed western blotting to quantify the proportions of AKT, phosphorylated AKT (p-AKT), PI3K, and phosphorylated PI3K (p-PI3K). In contrast with OM, DEX suppressed PI3K and AKT phosphorylation as illustrated by Western blotting. In contrast, epimedin C promoted the phosphorylation of PI3K and AKT in MC3T3-E1 cells subjected to DEX treatment on day 3 (Figure 5).

### LY294002 Suppressed the Protection Function of Epimedin C in MC3T3-E1 Cells Treated With DEX

**FIGURE 6:**
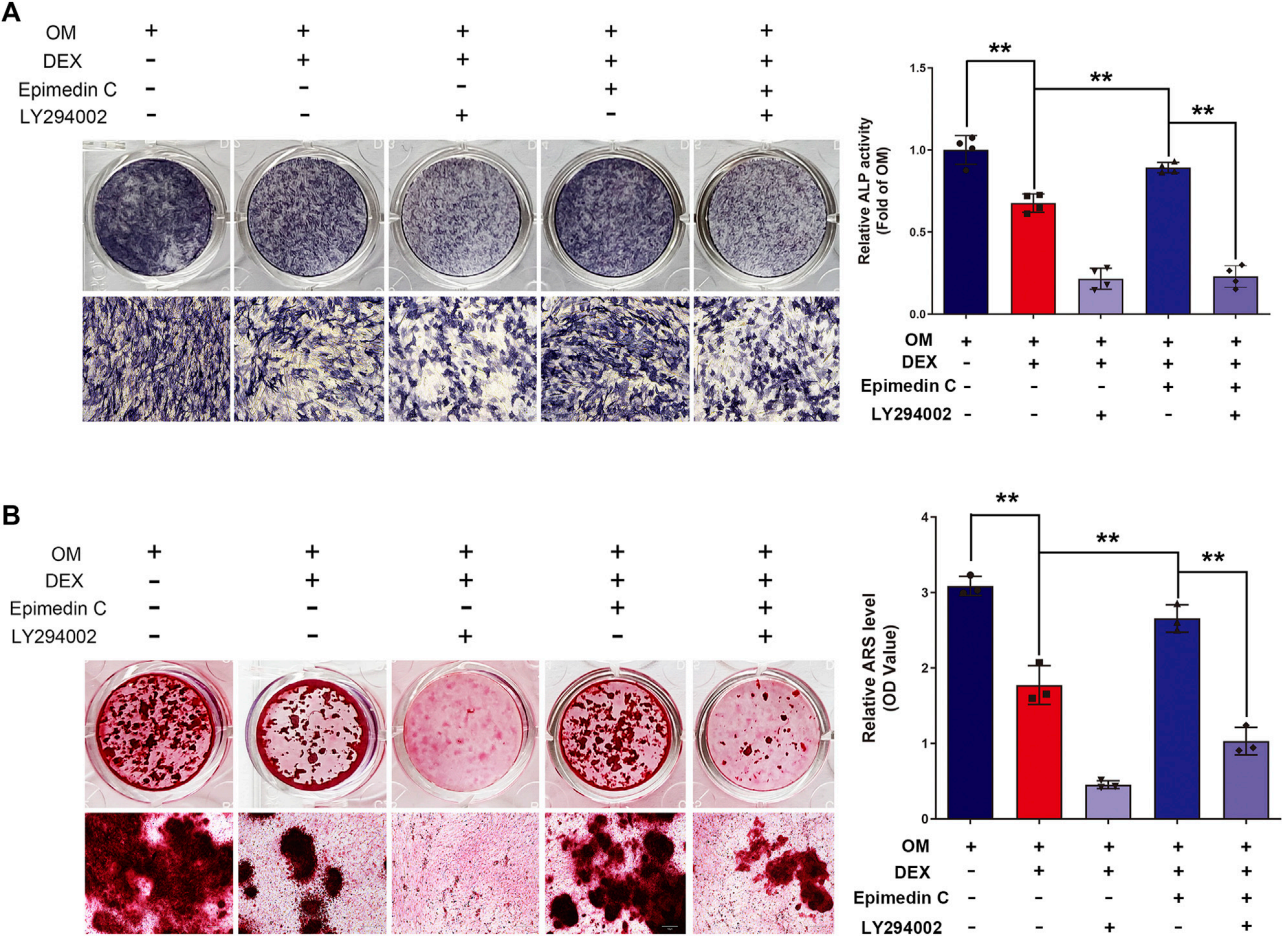
In MC3T3-E1 cells treated with DEX, LY294002 decreased epimedin C’s protective activity. **(A)** After 5 days of treatment with LY294002, DEX, and epimedin C, the MC3T3-E1 cells were stained with ALP with the aid of the BCIP/NBT kit. **(B)** Following 32 days of treatment using LY294002, DEX, and epimedin C, the mineralized nodules were subjected to staining with alizarin red S. DEX, dexamethasone; OM, osteogenic induction medium. ^**^ *p* < 0.01.

To additionally examine the function of PI3K and AKT signaling pathway in the protection functions of epimedin C, cells were subjected to treatment using PI3K inhibitor (LY294002), DEX, and epimedin C. Epimedin C (20 μM) considerably promoted the ALP function and mineralization nodes in MC3T3-E1 cells that had been treated using DEX in contrast with the DEX group (*p* < 0.01). However, the use of LY294002 significantly inhibited ALP activity and mineralized nodules in MC3T3-E1 cells (*p* < 0.01) (Figure 6).

### DEX-Mediated Skull Mineralization Decrease in Zebrafish Was Attenuated by Epimedin C

**FIGURE 7:**
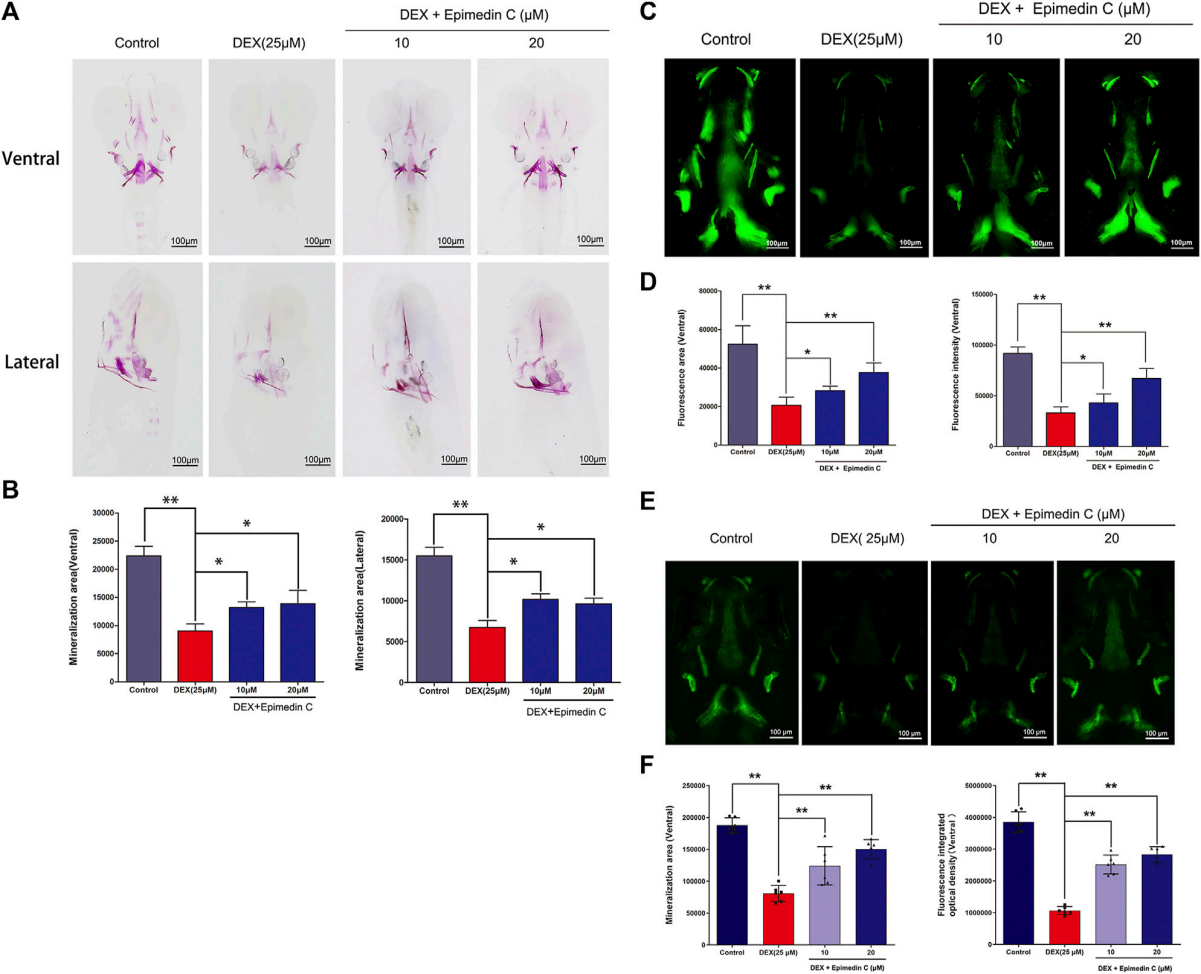
In zebrafish larvae, epimedin C reduced DEX-induced mediated reductions in skull mineralization. **(A)** To determine the magnitude of skull mineralization, the zebrafish larvae within the DEX, and DEX + epimedin C (10 and 20 μM), and control groups were stained with alizarin red S. **(B)** IPP was employed to examine the region of skull mineralization stained with alizarin red S. **(C)** To assess the extent of skull mineralization, zebrafish larvae within the DEX, and DEX + epimedin C (10 and 20 μM), and control groups were subjected to staining using calcein. **(D)** IPP was utilized to investigate the fluorescence region of skull mineralization stained with calcein. **(E,F)**: Zebrafish TG (ola.sp7:nlsGFP) treated with DEX and DEX + epimedin C (10 and 20 μM) and photographed using a fluorescence microscope. ^*^ *p < 0.05,* ^**^ *p < 0.01.*

Mineralization of the bones is an essential marker of bone development. The development of zebrafish bones was studied utilizing alizarin red S and calcein staining. The extent of mineralization of the skull can be assessed by performing alizarin red S and calcein with area staining. The DEX group’s bone mineralization region of the skull was clearly suppressed, according to the findings. In zebrafish, the administration of epimedin C (10 and 20 μM) ameliorated the impacts of DEX on the reduction in the mineralization area of bones in the skull (Figures 7A–D). Bone mass and density can be readily assessed by eGFP signal in zebrafish using TG (ola.sp7:nlsGFP). Morphological analysis of zebrafish TG (ola.sp7:nlsGFP) images showed that the signal intensity of green fluorescence in DEX-treated larvae showed a downward trend, and the mineralized area and IOD of larvae were significantly reduced. Epimedin C can significantly alleviate this inhibitory effect caused by dexamethasone (Figures 7E, F).

### LY294002 Suppressed Epimedin C’s Protection Impact on DEX-Mediated Skull Mineralization Decrease in Zebrafish

**FIGURE 8:**
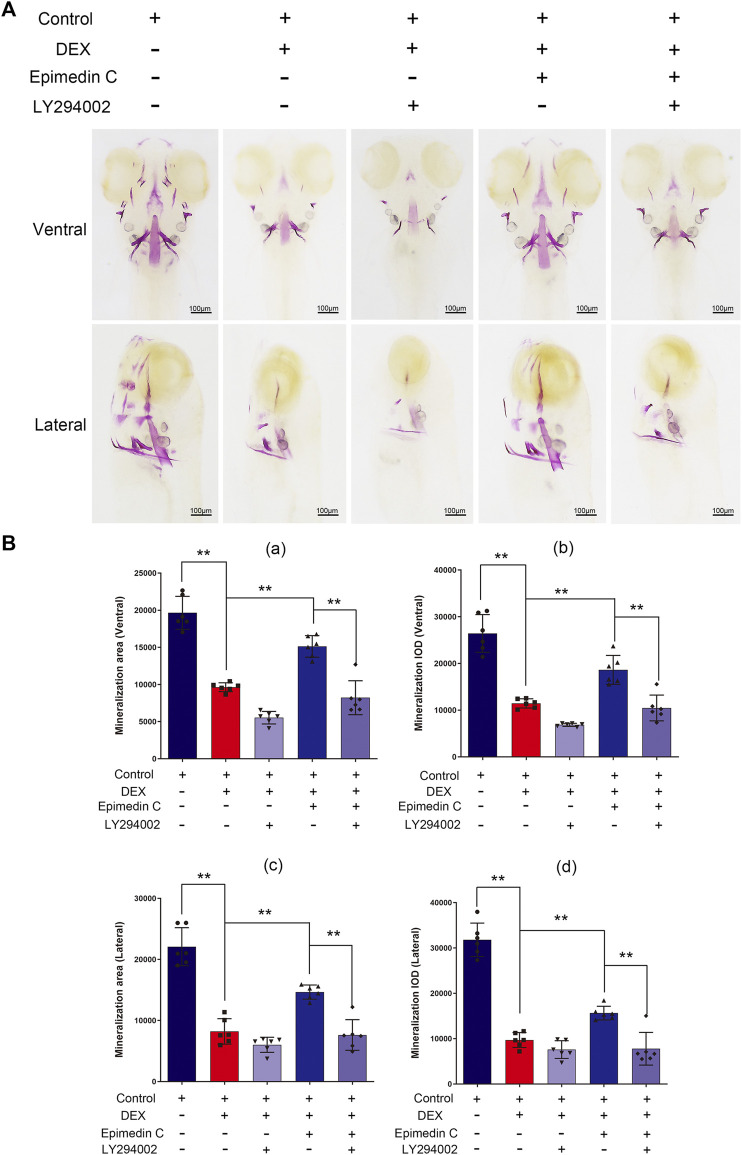
In zebrafish, LY294002 suppressed epimedin C’s protection function in DEX-mediated decreases in skull mineralization. **(A)** To estimate the extent of skull mineralization, the zebrafish larvae within the DEX, DEX + Epimedin C (20 μM), control, and DEX + Epimedin C + LY294002 groups were stained with alizarin red S. **(B)** IPP was used to examine the region of skull mineralization staining with alizarin red S.^**^ *p < 0.01.*

To subsequently examine the involvement of PI3K and AKT signaling pathway in the protection impacts of epimedin C, Zebrafish were subjected to treatment with PI3K inhibitor (LY294002), DEX, and epimedin C. The results illustrated that epimedin C (20 μM) considerably increased the integrated optical density (IOD) and mineralization area of skull, compared with the DEX group (*p* < 0.01). In DEX-mediated reduction of skull mineralization in Zebrafish, however, the introduction of LY294002 greatly suppressed the IOD and mineralization area of the skull elevated by epimedin C (*p* < 0.01) (Figure 8).

## Discussion

Glucocorticoid-induced osteoporosis (GIOP) is the most frequent secondary contributor to osteoporosis, resulting in severe morbidity. For the treatment of osteoporosis, postmenopausal patients with osteoporosis should choose estrogen therapy, however, it is not advised to utilize it for an extended period of time (Fait, 2019). Bisphosphonates, including alendronate and zoledronic acid, are by far the most frequently utilized agents for treating osteoporosis (Qaseem et al., 2017). Bisphosphonates, on the other hand, pose the danger of osteonecrosis of the jaw as well as atypical femoral fractures, which must not be overlooked (Adler et al., 2016). Based on the theory of traditional Chinese medicine and the use of Chinese herbal medicines as potential therapeutic strategies for osteoporosis is a growing area of research (An et al., 2016; Zhang et al., 2016). The beneficial properties of Chinese herbal medicine in clinical and experimental osteoporosis therapy are currently supported by published research and clinical studies (Zhang et al., 2016). Multiple research reports have demonstrated that Chinese herbal medication could help individuals with GIOP elevate their bone density and relieve their clinical symptoms (Zhang et al., 2021). Epimedii herba is a natural chemical that is progressively being explored in the treatment and prevention of osteoporosis due to its pharmacological properties of reducing bone resorption and accelerating bone formation (Ming et al., 2013). Epimedin C and icariin from epimedii herba, can enhance ALP activity, mineralization and promote osteoblast proliferation (Huang et al., 2019). Nevertheless, the pharmacological effects and mechanisms of epimedin C in GIOP are still to be studied. In this research, the pharmacological properties and mechanisms of epimedin C on suppressing the DEX-mediated osteogenesis in MC3T3-E1 cells and zebrafish were investigated.

Alkaline phosphatase is a well-known indicator of early bone formation and performs an instrumental function in bone growth (Harrison et al., 1995). Cell mineralization nodules are amongst the most significant indications of the later phase of osteogenic differentiation in view of the fact that they develop in the advanced stages of osteogenic differentiation to osteoblasts, accounting for around 2/3 of the osteogenic stage (Satomura and Nagayama, 1991; Hoemann et al., 2009). In MC3T3-E1 cells subjected to DEX treatment, the impact of epimedin C on the activity of ALP and mineralization nodules was investigated. The findings of ALP staining and ALP activity showed that exposure to DEX remarkably suppressed the ALP function in MC3T3-E1 cells. Contrastingly, the inhibited ALP activity was significantly alleviated when epimedin C was present. We found that DEX inhibited mineralization nodes of MC3T3-E1 cells compared with OM. Nevertheless, the inhibited effect was significantly alleviated when epimedin C was added. These findings demonstrated that epimedin C reduced the inhibitory activity of DEX on osteogenic differentiation.

RUNX2, a constituent of the Runx family of transcriptional factors, is essential for osteoblast differentiation, bone production, and chondrocyte maturation (Satomura and Nagayama, 1991; Hoemann et al., 2009). Osterix (OSX or Sp7) is a transcriptional factor belonging to the zinc finger family that is considered necessary for osteoblast differentiation (Nakashima et al., 2002). OSX is suppressed in all growing bones, and Osx-null mice have no bone development. The expression of OSX is modulated by RUNX2, the prevalent upstream transcriptional factor required for osteoblast differentiation (Komori, 2017). Studies have shown that osteoblast differentiation could be enhanced by stimulating OSX and RUNX2 (Choi et al., 2016; Choi et al., 2018). Earlier studies of our group have confirmed that dexamethasone can significantly inhibit the expression of transcription factors OSX and RUNX2 (Xie et al., 2021). The findings illustrated that the DEX group had considerably lower OSX and RUNX2 protein expression levels, whereas epimedin C mitigated the antagonistic function of DEX and dramatically enhanced OSX and RUNX2 protein expression in contrast with the DEX group. ALPL is also known as tissue non-specific alkaline phosphatase, and deficiency of this protein results in loss of bone and tooth mineralization (Millán and Whyte, 2016). Alpl^(−/−)^ mice exhibit a craniofacial skeletal phenotype with severely diminished bone mineralization (Liu J et al., 2014). This research illustrated that DEX could inhibit the expression of ALPL protein, and epimedin C could reverse the suppressive impact of DEX and promote the expression of ALPL protein. ALPL overexpression increases the expression of osteoblast transcription factor master genes RUNX2 and OSX, as well as mature osteoblast and osteocyte marker genes (Nakamura et al., 2020). Therefore, we believe that epimedin C attenuated the suppressive function of DEX on osteogenic differentiation and promotes osteogenic differentiation is closely related to the regulatory relationship between RUNX2, OSX, and ALPL.

In osteoporosis, the PI3K/Akt signaling cascade is triggered, which performs a fundamental function in the osteoclast’s hyperactivation as well as the differentiation and maturation of osteoblasts (Han et al., 2021). Astragaloside positively regulated osteogenic differentiation of MC3T3-E1 by triggering the PI3K/Akt signaling pathway (Jing et al., 2021). Research has found that dexamethasone inhibits the osteogenic differentiation of *osteoblasts in vivo* and *in vitro* via the PI3K/Akt signaling pathway (Pan et al., 2019). DEX suppressed the PI3K and AKT phosphorylation, according to the results of our research. Contrastingly, epimedin C promoted phosphorylation of PI3K and AKT in MC3T3-E1 cells subjected to DEX treatment. By stimulating the PI3K/Akt signaling pathway, we believe that epimedin C could attenuate glucocorticoid-mediated suppression of osteogenic differentiation. To further confirm our hypothesis, we observed ALP activity and mineralized nodule formation after inhibition with a PI3K inhibitor (LY294002). We found that epimedin C considerably elevated the ALP function and mineralization nodes in MC3T3-E1 cells subjected to DEX treatment. On the other hand, the presence of LY294002 remarkably suppressed the activity of ALP and mineralization nodes enhanced by epimedin C in MC3T3-E1 cells treated with DEX. This revealed that epimedin C could alleviate glucocorticoid-mediated suppression of osteogenic differentiation via the PI3K/Akt signaling pathway activation.

The zebrafish is a feasible model organism that has been used in scientific research with great success. The zebrafish larvae are highly transparent, making it possible to readily observe their bone morphology. As a result, zebrafish might serve as an excellent model organism for investigating bone growth (Carnovali et al., 2019; Marí-Beffa et al., 2021). The most common way to study zebrafish bone models is through classic bone staining and observation under a microscope. Common chemical dyes used in this model include alizarin Red S and calcein (Luo et al., 2019). We employed alizarin red S and calcein to visualize the bone development of zebrafish in this research. The extent of the skull mineralization area may be measured by staining with alizarin red S and calcein. The introduction of epimedin C to DEX attenuated the impact of DEX on the reduction of bone mineralization area of the skull in zebrafish. Zebrafish were administered with LY294002, DEX, and epimedin C to better comprehend the role of the PI3K/AKT signaling pathway in epimedin C’s protective impact. In comparison with the DEX group, epimedin C remarkably enhanced the mineralization area and IOD of the skull. But, the addition of LY294002 significantly inhibited the IOD and mineralization area of skull increased by epimedin C in DEX-mediated reduction of skull mineralization in Zebrafish. Our results showed that epimedin C attenuated the suppressive impact of DEX in the osteogenesis of zebrafish larval by triggering the PI3K and AKT signaling pathways. Osteoclasts and osteoblasts are not exist alone, while communicating with each other through direct contact, diffusible paracrine factors and cell-bone matrix interaction. Flavonoids from Herba Epimedii have been shown to have therapeutic effect against bone loss. The study showed that Icariside II inhibited pre-osteoclast RAW264.7 growth. Icaritin, another flavonoid constituent, was shown here to inhibit RAW264.7 growth in a dose-dependent manner (Liu YQ et al., 2014). We have not found any related research on epimedin C on osteoclasts, this is our follow-up research direction.

## Conclusion

The current research demonstrated the involvement of epimedin C in alleviating the antagonistic activity on osteogenic differentiation mediated by DEX via the mechanism of triggering the PI3K/AKT signaling pathway. Overall, our findings illustrated that epimedin C may be a promising option for development as a novel treatment approach for GIOP.

## Data Availability

The original contributions presented in the study are included in the article/Supplementary Material further inquiries can be directed to the corresponding authors.
