# Supplementary material for: Epimedin C Alleviates Glucocorticoid-Induced Suppression of Osteogenic Differentiation by Modulating PI3K/AKT/RUNX2 Signaling Pathway
Source: Front Pharmacol. 2022 Jul 4;13:894832. doi: 10.3389/fphar.2022.894832 (PMC9291512; doi:10.3389/fphar.2022.894832)
Supplement: Supplementary file 1 [file Table1.DOCX]

Raw data link 2022.06.06

https://www.jianguoyun.com/p/DTBlMe8QzaXTChijlcUEIAA
